# γδ T Cells Are Required for M2 Macrophage Polarization and Resolution of Ozone-Induced Pulmonary Inflammation in Mice

**DOI:** 10.1371/journal.pone.0131236

**Published:** 2015-07-02

**Authors:** Joel A. Mathews, David I. Kasahara, Luiza Ribeiro, Allison P. Wurmbrand, Fernanda M. C. Ninin, Stephanie A. Shore

**Affiliations:** Molecular and Integrative Physiological Sciences Program, Department of Environmental Health, Harvard T.H. Chan School of Public Health, Boston, Massachusetts, United States of America; University of Alabama at Birmingham, UNITED STATES

## Abstract

We examined the role of γδ T cells in the induction of alternatively activated M2 macrophages and the resolution of inflammation after ozone exposure. Wildtype (WT) mice and mice deficient in γδ T cells (TCRδ^-/-^ mice) were exposed to air or to ozone (0.3 ppm for up to 72h) and euthanized immediately or 1, 3, or 5 days after cessation of exposure. In WT mice, M2 macrophages accumulated in the lungs over the course of ozone exposure. Pulmonary mRNA abundance of the M2 genes, *Arg1*, *Retnla*, and *Clec10a*, also increased after ozone. In contrast, no evidence of M2 polarization was observed in TCRδ^-/-^ mice. WT but not TCRδ^-/-^ mice expressed the M2c polarizing cytokine, IL-17A, after ozone exposure and WT mice treated with an IL-17A neutralizing antibody exhibited attenuated ozone-induced M2 gene expression. In WT mice, ozone-induced increases in bronchoalveolar lavage neutrophils and macrophages resolved quickly after cessation of ozone exposure returning to air exposed levels within 3 days. However, lack of M2 macrophages in TCRδ^-/-^ mice was associated with delayed clearance of inflammatory cells after cessation of ozone and increased accumulation of apoptotic macrophages in the lungs. Delayed restoration of normal lung architecture was also observed in TCRδ^-/-^ mice. In summary, our data indicate that γδ T cells are required for the resolution of ozone-induced inflammation, likely because γδ T cells, through their secretion of IL-17A, contribute to changes in macrophage polarization that promote clearance of apoptotic cells.

## Introduction

Exposure to the air pollutant, ozone (O_3_), has a significant impact on human health. O_3_ exposure causes respiratory symptoms, reductions in lung function, and may even increase the risk of mortality in those with preexisting lung disease [1,2,3,4,5,6]. O_3_ causes oxidative stress and subsequent damage to lung and airway epithelial cells, leading to the production of numerous cytokines and chemokines, and recruitment of neutrophils and macrophages to the lungs [1,7]. In WT mice, the resolution of inflammation and injury occurs within 72 hours of cessation of subacute O_3_ exposure (0.3 ppm for 72 h) [8], though some effects of O_3_ persist even 72 h after a more prolonged exposure [9]. While the processes promoting O_3_-induced inflammation are relatively well understood, the processes that control the resolution of O_3_-induced inflammation are not. Nevertheless, termination of O_3_-induced inflammation and repair of damaged lung cells is key to protecting the lung from the cytotoxic effects of inflammatory cells and mediators.

Alternatively activated M2 macrophages have the capacity to phagocytose apoptotic cells and debris from necrotic cells, and participate in the resolution and repair of tissue damage induced by a variety of agents [10,11]. For example, M2 macrophages contribute to epithelial tubular cell repair after ischemic renal injury [12]. M2 macrophages, particularly M2c macrophages, are also required for clearance of apoptotic neutrophils and macrophages [13,14]. M2 macrophages are observed in the lungs after acute high dose O_3_ exposure in mice [15,16], but whether such cells are present in the lungs after a lower concentration, but longer duration of O_3_ exposure has not been established.

γδ T cells compose part of the innate immune system and are found primarily in non-lymphatic organs, including the lung [17]. γδ T cells contribute to inflammatory cell recruitment in response to many types of injury and infection, both in the lungs and in other tissues [18,19,20,21,22,23]. However, γδ T cells also participate in the resolution of injury and inflammation. For example, γδ T cells are important for wound repair in the skin [24]. In the lung, γδ T cells are required for the resolution of eosinophilic inflammation after allergen challenge [25] and for the resolution of macrophage infiltration after *S*. *pneumonia* infection [26]. The role of γδ T cells in the resolution of pulmonary injury and inflammation after subacute O_3_ exposure has not been established, but could be important.

Since IL-17A promotes M2c polarization [13], γδ T cells could contribute to resolution of O_3_-induced injury and inflammation via their capacity to produce IL-17A. We have established that pulmonary *Il17a* mRNA abundance increases after O_3_ exposure and that O_3_ increases the number of IL-17A^+^ γδ T cells in the lungs [27,28]. Furthermore, γδ T cells are required for expression of IL-17A after subacute ozone [28]: O_3_-induced increases in pulmonary *Il17a* mRNA are observed in wildtype (WT) mice but not in mice lacking γδ T cells (TCRδ^-/-^ mice). The purpose of this study was to examine the hypothesis that γδ T cells contribute to M2 macrophage polarization and the resolution of inflammation and injury after subacute O_3_ exposure in mice. To test this hypothesis, we assessed lung M2 macrophages and M2 gene expression by flow cytometry and RT-qPCR, respectively, during and after exposure of mice to O_3_ (0.3 ppm for up to 72 h). Experiments were performed both in WT and TCRδ^-/-^ mice. We also performed bronchoalveolar lavage (BAL) in order to examine the clearance of inflammatory cells and mediators recruited to lungs by O_3_ exposure. Finally, we used flow cytometry to examine the apoptotic status of macrophages after cessation of O_3_ exposure. Our results indicate the γδ T cells are required for M2 macrophage polarization after subacute O_3_ exposure, likely as a result of the ability of γδ T cells to produce IL-17A. Moreover, the absence of M2 macrophages in γδ T cell deficient mice was associated with delayed clearance of inflammatory cells and retention of apoptotic macrophages in the lungs of these mice after cessation of O_3_ exposure.

## Methods

### Animals

This study was approved by the Harvard Medical Area Standing Committee on Animals. Male age-matched WT and TCRδ^-/-^ mice were bred in house from breeding pairs originally purchased from The Jackson Laboratory (Bar Harbor, ME). All mice were on a C57BL/6J background, fed a standard mouse chow diet, and were 10–13 weeks old at the time of study.

### Protocol

Mice were exposed to room air for 48 h or to O_3_ (0.3 ppm) for 24, 48 or 72 h and euthanized immediately after exposure with an overdose of sodium pentobarbital. These mice were previously described [28]. Other mice were exposed to O_3_ (0.3 ppm) for 72 hours, allowed to recover in room air, and euthanized 1, 3, or 5 days after cessation of exposure. Tissue and BAL were then collected and analyzed as previously described [27,28]. In another cohort of mice, whole lungs were processed for flow cytometry to examine macrophage apoptosis. BAL was not performed on these mice so that we could examine both alveolar and interstitial macrophages for evidence of apoptosis. The protocols used for anti-IL-17A treatment were previously described [27,28].

### Ozone exposure

During O_3_ exposure, mice were placed in their regular home cages with the microinsulator lids removed. Cages were placed inside stainless steel and Plexiglas exposure chambers and exposed as described previously [27]. Mice had free access to normal chow and to water during exposure.

### Bronchoalveolar lavage

BAL was performed and cells counted as previously described [27]. BAL supernatant was stored at −80°C until assayed for G-CSF and MCP1 by ELISA (R&D Systems) and TNFα by ELISA (eBioscience San Diego, CA). Total BAL protein was measured by Bradford assay (Bio-Rad, Hercules, CA).

### Flow cytometry

The left lung was harvested and placed on ice in RPMI 1640 media containing 2% FBS and HEPES. Lungs were digested, prepared for flow cytometry, and analyzed as previously described [27,28]. For M1/M2 macrophage analysis the following antibodies were used: Alexa Fluor 488 anti-F4/80 (clone: BM8), PE—anti-CD206 (clone: C068C2), Percp/cy5.5- anti-CD80 (Clone: 16-10A1). For macrophage apoptosis staining, the whole lung (without bronchoalveolar lavage) was used and single cell suspension was stained with the following antibodies: PE-cy7 anti-F4/80, PE—anti CD11c (clone: N418), 7-AAD, and FITC anti-Annexin V.

### Real-time PCR

RNA was extracted from lung tissue and cDNA prepared for qPCR as previously described [27]. The primers for *Il17a*, *Rplp0*, *Cldn4*, *Clec10a (Mgl1)*, *Retnla* and *Il13* were all previously described [29,30,31,32]. In addition, the following primers were used: *Arg1* forward: GTGTACATTGGCTTGCGAGA; reverse: GGTCTCTTCCATCACCTTGC. Melting curves yield a single peak for each primer; *Ym1* forward: GAA GGA GCC ACT GAG GTC TG; reverse: TTG TTG TCC TTG AGC CAC TG; *Mrc1* forward: CAA GGA AGG TTG GCA TTT GT; reverse: CAA GGA AGG TTG GCA TTT GC Expression values were normalized to *Rplp0* expression using the ΔΔCt method.

### Histology

Lungs were fixed with 4% paraformaldehyde under 20 cm of pressure for 1 min. The mainstem bronchus was then tied off. The lung was removed and placed overnight in a 50 ml conical containing 4% paraformaldehyde. Lungs were then transferred to tubes containing 70% ethanol. Lungs were sliced, first sagittally and then transversely. Slices were embedded in paraffin, sectioned, and stained with hematoxylin and eosin by the Rodent Histology Core (Harvard Medical School, Boston, MA). Histological examination of sections from O_3_-exposed mice indicated interstitial expansion of mononuclear cells and hyperplasia of epithelial cells in the region of terminal bronchioles. The slides were blinded and then each terminal bronchiole was scored for the number of cellular layers below the epithelium using the following scoring system: 0 for no lesions, 1: 1–2 cells, 2: for 3 cells, 3: for 4 cells, and 4: for 5 cells or more. At least 8 terminal bronchioles were scored in each mouse and the scores averaged to obtain a total lesion score for each mouse.

### Statistical analysis

ANOVA or factorial ANOVA using STATISTICA software (Statistica, StatSoft; Tulsa, OK) was used to analyze the data with either genotype and duration of time post exposure or just duration of time post exposure as main effect. To examine the effects of anti-IL-17A on M2 gene expression, factorial ANOVA using antibody treatment and exposure time (48 or 72 h) was used. A *p* value <0.05 was considered significant.

## Results

### Subacute ozone exposure induces M2 macrophage polarization in WT but not TCRδ^-/-^ mice

**Fig 1:**
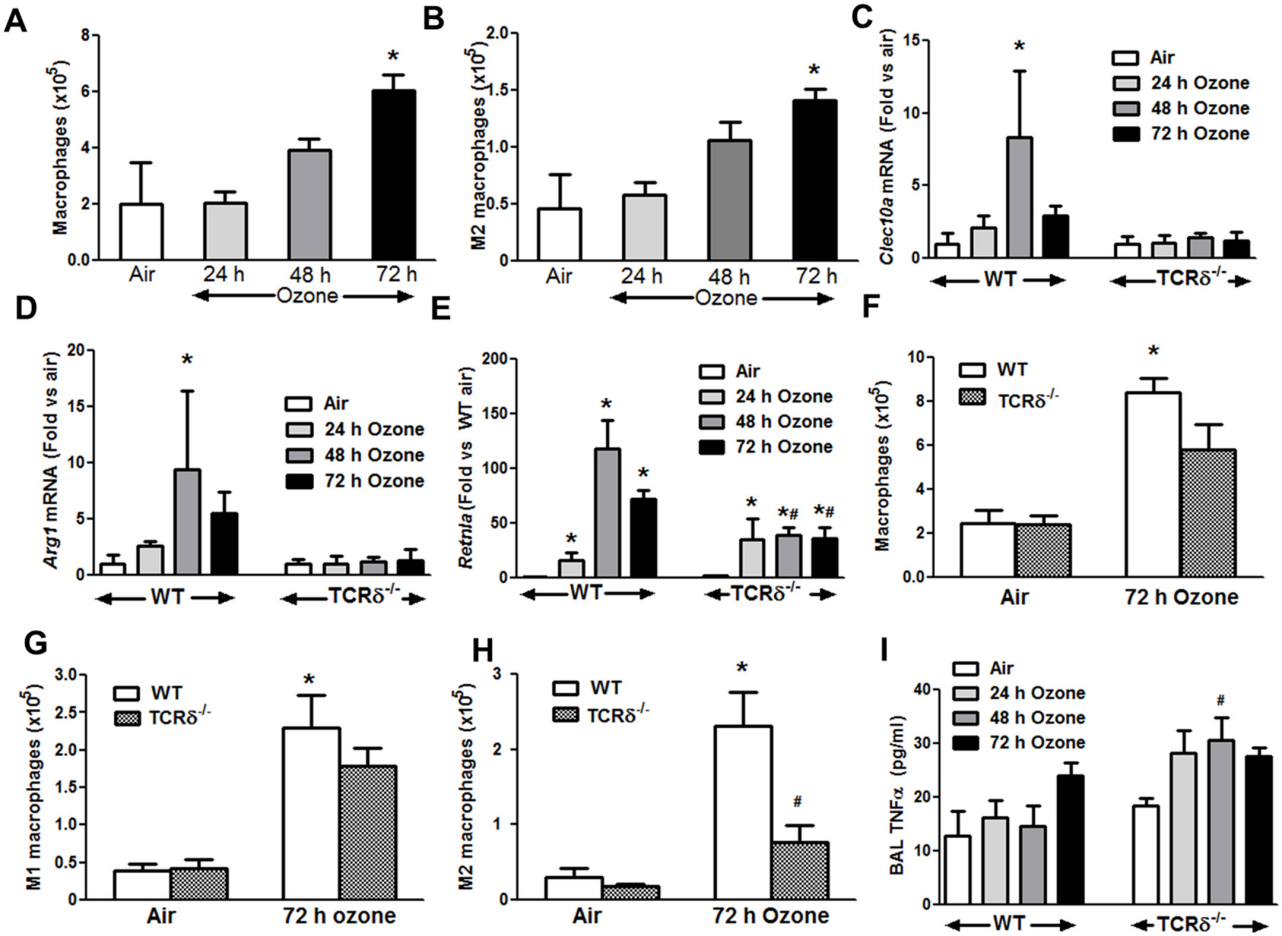
Induction of M2 macrophage by subacute O_3_ exposure is reduced in TCRδ^-/-^ mice. WT mice were exposed to either air or O_3_ (0.3 ppm) for 24, 48 or 72 hours and euthanized immediately after exposure. (A) Total lung macrophages (F4/80^+^ cells) and (B) total lung M2 macrophages (F4/80^+^CD80^-^CD206^+^ cells) were measured by flow cytometry. The pulmonary mRNA abundance of M2 markers (C) *Arg1* (D) *Clec10a* and (E) *Retnla* were also assessed by RT-qPCR in WT and TCRδ^-/-^ mice exposed to room air or O_3_. Total macrophages (F), M1 macrophages (G) and M2 macrophages (H) were also assessed in WT and TCRδ^-/-^ mice exposed to air or O_3_ (0.3 ppm for 72 h). (I) BAL TNFα was measured in the BAL by ELISA. Results are mean ± SE of 4–8 air exposed mice and 6–14 O_3_ exposed mice in each group. * p<0.05 versus air; # p<0.05 versus WT mice.

Total macrophages (F4/80^+^ cells) and M2 macrophages (F4/80^+^CD206^+^CD80^-^ cells) were measured by flow cytometry in lungs of WT mice exposed to air or to ozone (0.3 ppm) for 24, 48 or 72 hours and studied immediately after cessation of exposure. O_3_ caused a time dependent increase in total lung macrophages (Fig 1A), and in M2 macrophages (Fig 1B). For M2 macrophages, the peak occurred after 72 hours of exposure. In WT mice, the pulmonary mRNA abundances of *Arg1*, *Clec10a*, *and Retnla*, markers of M2 polarization [33,34], were also increased after O_3_ exposure (Fig 1C, 1D and 1E). O_3_-induced increases in total lung macrophages and M1 macrophages (F4/80^+^CD206^-^CD80^+^) were not affected by γδ T cell deficiency (Fig 1F), but M2 macrophages were reduced in O_3_ exposed TCRδ^-/-^ versus WT mice (Fig 1G and 1H). We also observed no induction of the M2 macrophage markers, *Arg1* and *Clec10a*, in TCRδ^-/-^ mice after O_3_ (Fig 1C and 1D), *Retnla* mRNA was induced by O_3_ in TCRδ^-/-^ mice (Fig 1E). However, compared to WT mice, in TCRδ^-/-^ mice levels of *Retnla* were significantly lower after 48 and 72 h of exposure consistent with decreased M2 macrophages. Of note, *Retnla* is also highly expressed in epithelial cells [35], and the *Retnla* mRNA observed in O_3_-exposed TCRδ^-/-^ mice (Fig 1E) may derive from epithelial cells rather than M2 macrophages. To determine whether the decrease in M2 macrophages in TCRδ^-/-^ mice was associated with increased activity of M1 macrophages, we measured BAL TNFα (Fig 1I): TNFα is predominately expressed by M1 macrophages [36]. BAL TNFα was higher in the TCRδ^-/-^ versus WT mice after 48 hours of O_3_ exposure, the point where gene express for M2 macrophages peaked in WT mice (Fig 1C to 1E).

**Fig 2:**
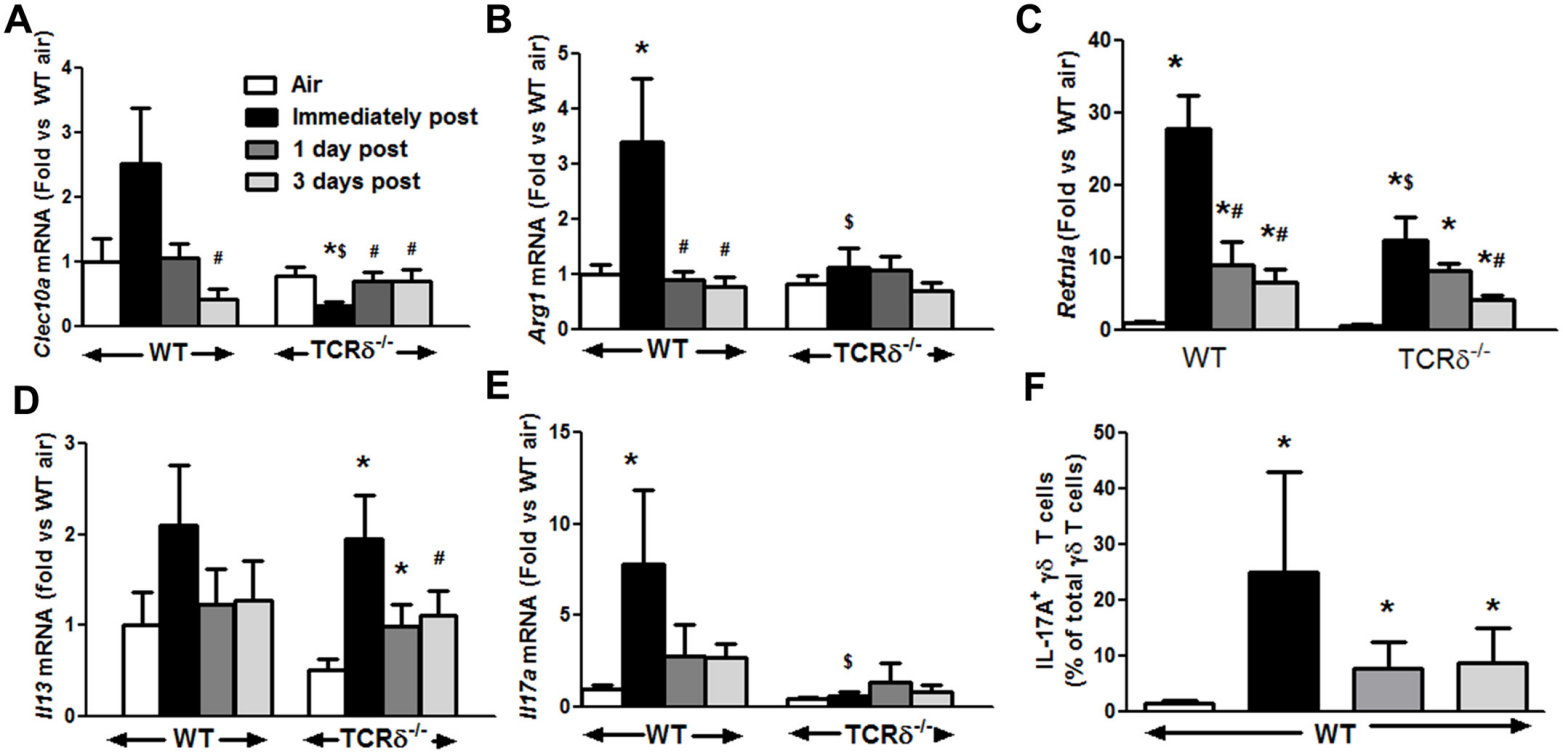
Pulmonary M2 gene expression after cessation of O_3_ exposure. Pulmonary (A) *Clec10a*, (B) *Arg1*, (C) *Retnla*, (D) *Il13*, *and (E) Il17a* mRNA abundance in WT and TCRδ^-/-^ mice exposed to room air or to ozone (O_3_, 0.3 ppm for 72 h) and then euthanized either immediately or 1 or 3 days after cessation of O_3_ exposure. (F) IL-17A^+^γδ were determined by flow cytometry. Note that data from the air and immediately post mice have been previously published [28] Results are mean ± SE of 4–8 air exposed mice and 6–14 O_3_ exposed mice in each group. * p<0.05 versus air; # p<0.05 versus 72 hour O_3_; $ p<0.05 versus WT mice.

To determine the duration of elevations in M2 macrophages after cessation of O_3_ exposure, we measured the pulmonary abundance of *Arg1*, *Clec10a*, and *Retnla* in mice after air exposure, and immediately after or 1 or 3 days after cessation of O_3_ exposure (Fig 2A to 2C). In WT mice, the pulmonary mRNA abundances of *Arg1*, *Clec10a*, and *Retnla* were elevated immediately after cessation of O_3_ exposure, as described above. Both *Arg1* and *Clec10a* returned to air exposed levels within 1 day after cessation of exposure (Fig 2A and 2B). *Retnla* mRNA abundance also declined rapidly after cessation of O_3_, but was still elevated through day 3 (Fig 2C). In contrast, in TCRδ^-/-^ mice, *Arg1* and *Clec10a* were not induced at any time after cessation of O_3_ exposure (Fig 2A and 2B). As described above, *Retnla* levels were significantly lower in TCRδ^-/-^ mice than in WT mice immediately after cessation of O_3_, but resembled levels in WT thereafter, likely because the persistent *Retnla* expression derived from epithelial cells rather than M2 macrophages. IL-13 and IL-4 can induce M2 polarization [37], but microarray data from our lab indicates no changes in Il4 mRNA expression after O_3_ [32] O_3_-induced changes in pulmonary IL-13 mRNA abundance were similar in TCRδ^-/-^ and WT mice (Fig 2D).

**Fig 3:**
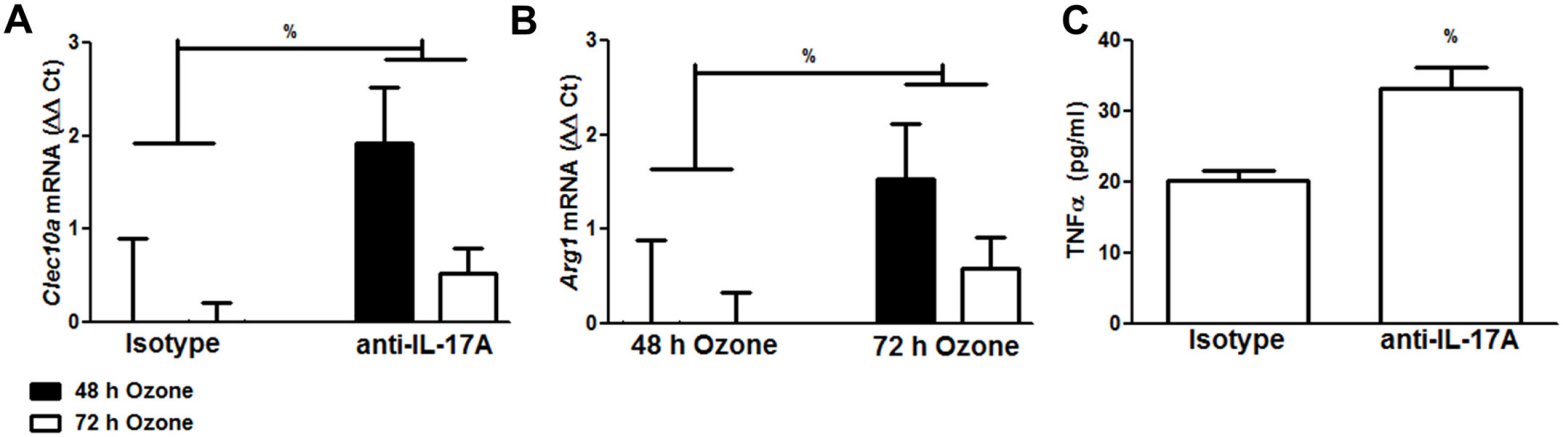
Blocking IL-17A reduces pulmonary expression of *Arg1 and Clec10a*. Pulmonary mRNA abundance of (A) *Clec10a* and (B) *Arg1* measured as changes in Ct values in lungs from mice treated with IL-17A neutralizing versus isotype control antibody injected i.p. prior to O_3_ exposure. Note that an increase in Ct indicates a decrease in expression. Mice were exposed to O_3_ for either 48 or 72 h and euthanized immediately after cessation of exposure. Other data from these mice has been previously published [27,28]. (C) As a marker of M1 activation, TNFα was measured in the BAL by ELISA. Results are mean ± SE 5–7 mice in each group. % p<0.05 versus isotype control, as assessed by factorial ANOVA.

IL-17A can also drive M2 macrophage polarization [13] and we have previously reported that pulmonary mRNA abundance of *Il17a* is increased after subacute O_3_ exposure in WT but not TCRδ^-/-^ mice [28]. RT-qPCR confirmed and extended these observations: in WT mice, pulmonary *Il17a* mRNA peaked immediately after cessation of O_3_ and then gradually resolved over the next 3 days, whereas no increase in *Il17a* mRNA abundance was observed in TCRδ^-/-^ mice at any time after cessation of O_3_ exposure (Fig 2E). We have also reported that in WT mice the number of IL-17A^+^γδ T cells increases with O_3_ exposure[27,28]. Flow cytometry indicated that the number of IL-17A^+^γδ T cells remained elevated in the WT mice until day 3 post exposure (Fig 2F). Consequently, we examined the hypothesis that the lack of M2 polarization in TCRδ^-/-^ mice after O_3_ exposure was the result of their inability to produce IL-17A. To do so, WT mice were treated with either isotype control antibody or with anti-IL-17A [27,28] prior to exposure and examined immediately after either 48 or 72 h of O_3_ exposure. Factorial ANOVA using exposure time (48 or 72 h) and treatment (isotype or anti-IL-17A) as main effects indicated a significant effect of treatment on mRNA expression of both *Arg1* and *Clecl10a* (Fig 3A and 3B) and that the effect lay in the animals exposed to O_3_ for 48 h, the peak of O_3_-induced changes in M2 gene expression (Fig 1C to 1E). Note that *Arg1* and *Clec10a* mRNA abundances were significantly lower in anti-IL-17A versus isotype treated mice (i.e. **ΔΔ**Ct values were higher). There was also a trend towards reduced expression of two other M2 genes, *Mrc1* and *Retnla*, at 48 hour of O_3_ exposure, but the effect did not reach statistical significance (data not shown). BAL levels of TNFα were also *increased* by anti-IL-17A treatment, indicating more M1 activation, similar to what was observed in TCRδ^-/-^ mice (Fig 1I).

### Role of γδ T cells in the resolution of O_3_-induced increases in BAL inflammatory cells

**Fig 4:**
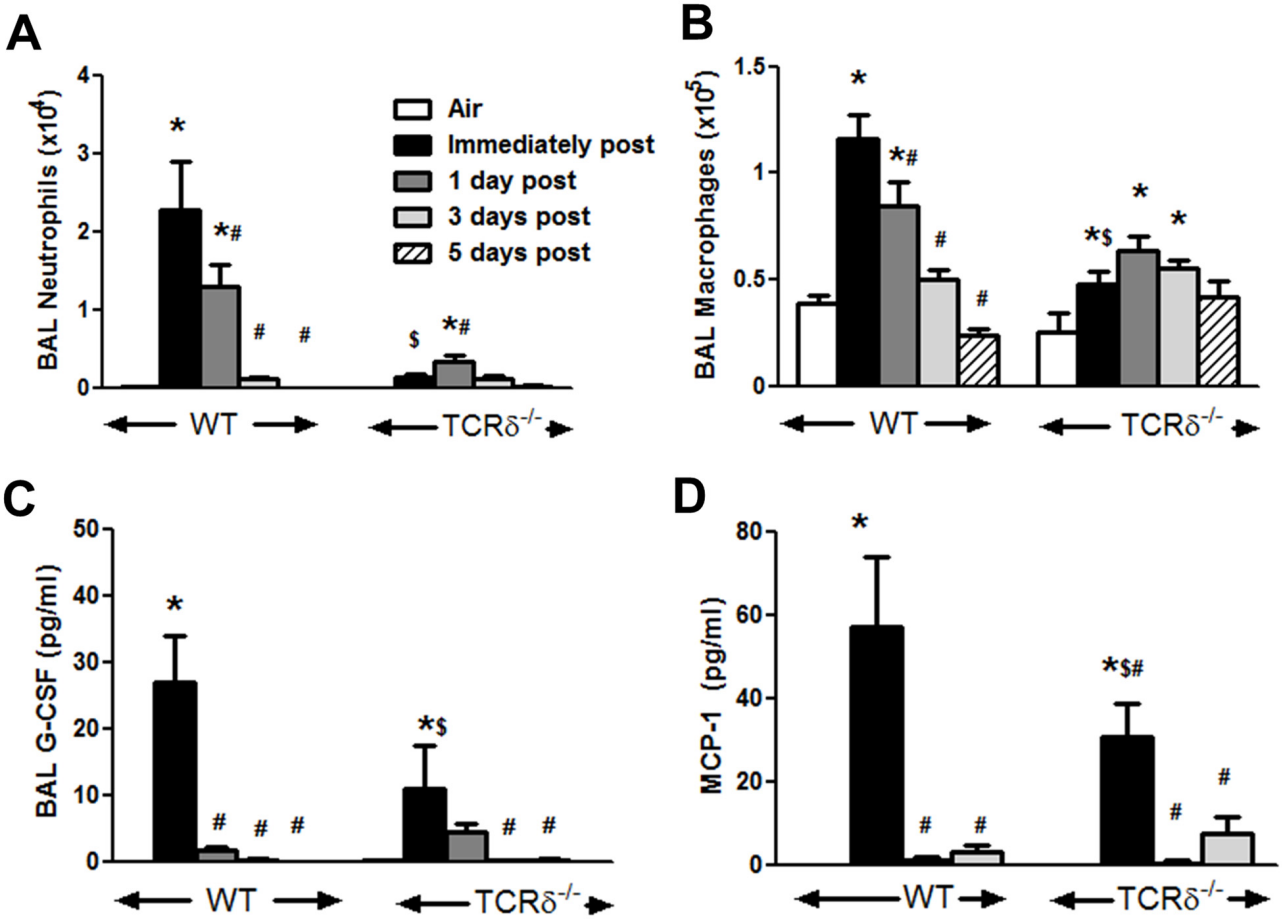
O_3_-induced inflammation in WT and γδ T cell deficient mice after cessation of O_3_ exposure. Bronchoalveolar lavage (BAL) neutrophils (A), macrophages (B), G-CSF (C), MCP-1 in wildtype (WT) and γδ T cell deficient (TCRδ^-/-^) mice exposed to room air or to ozone (O_3_, 0.3 ppm for 72 h) and then euthanized either immediately or 1, 3, or 5 days after cessation of O_3_ exposure. Data for the air and immediately post O_3_ time points have been previously published [28]. Results are mean ± SE of 4–8 air exposed mice and 6–14 O_3_ exposed mice in each group. * p<0.05 versus air; # p<0.05 versus immediately post O_3_; $ p<0.05 versus WT mice.

To determine if γδ T cells are required for resolution of O_3_-induced inflammation, mice were exposed to O_3_ (0.3 ppm) for 72 h and then allowed to recover in room air for 1, 3, or 5 days. Compared to air, BAL neutrophils and macrophages were significantly increased by O_3_ exposure (Fig 4A and 4B) in WT mice, consistent with previous reports by ourselves and others [28,32,38,39]. BAL neutrophils and macrophages declined rapidly thereafter, returning to values not significantly different from pre-exposure (air) values within 3 days of the cessation of O_3_ exposure (Fig 4A and 4B). In TCRδ^-/-^ mice, BAL neutrophils and macrophages were significantly lower than in WT mice immediately after cessation of O_3_ exposure (Fig 4A and 4B), as we have previously reported [28]. However, in contrast to WT mice, there was no reduction in either BAL neutrophils or BAL macrophages 1 day after versus immediately after cessation of exposure in TCRδ^-/-^ mice (Fig 4A and 4B). Indeed, in TCRδ^-/-^ mice, BAL neutrophils actually peaked not immediately after O_3_ exposure, as in the WT mice, but 1 day after cessation of exposure and began to decline thereafter (Fig 4A). In addition, in TCRδ^-/-^ mice, O_3_-induced elevations in BAL macrophages were sustained through 5 days after exposure (Fig 4B). This delayed clearance of inflammatory cells in TCRδ^-/-^ mice was not the result of more sustained increases in neutrophil and macrophage chemoattractant/survival factors in these mice: in both WT and TCRδ^-/-^ mice, BAL G-CSF and MCP-1 were induced by O_3_ but returned to levels not different from air exposed controls within 1 day of cessation of O_3_ exposure (Fig 4C and 4D).

### Macrophage apoptosis

**Fig 5:**
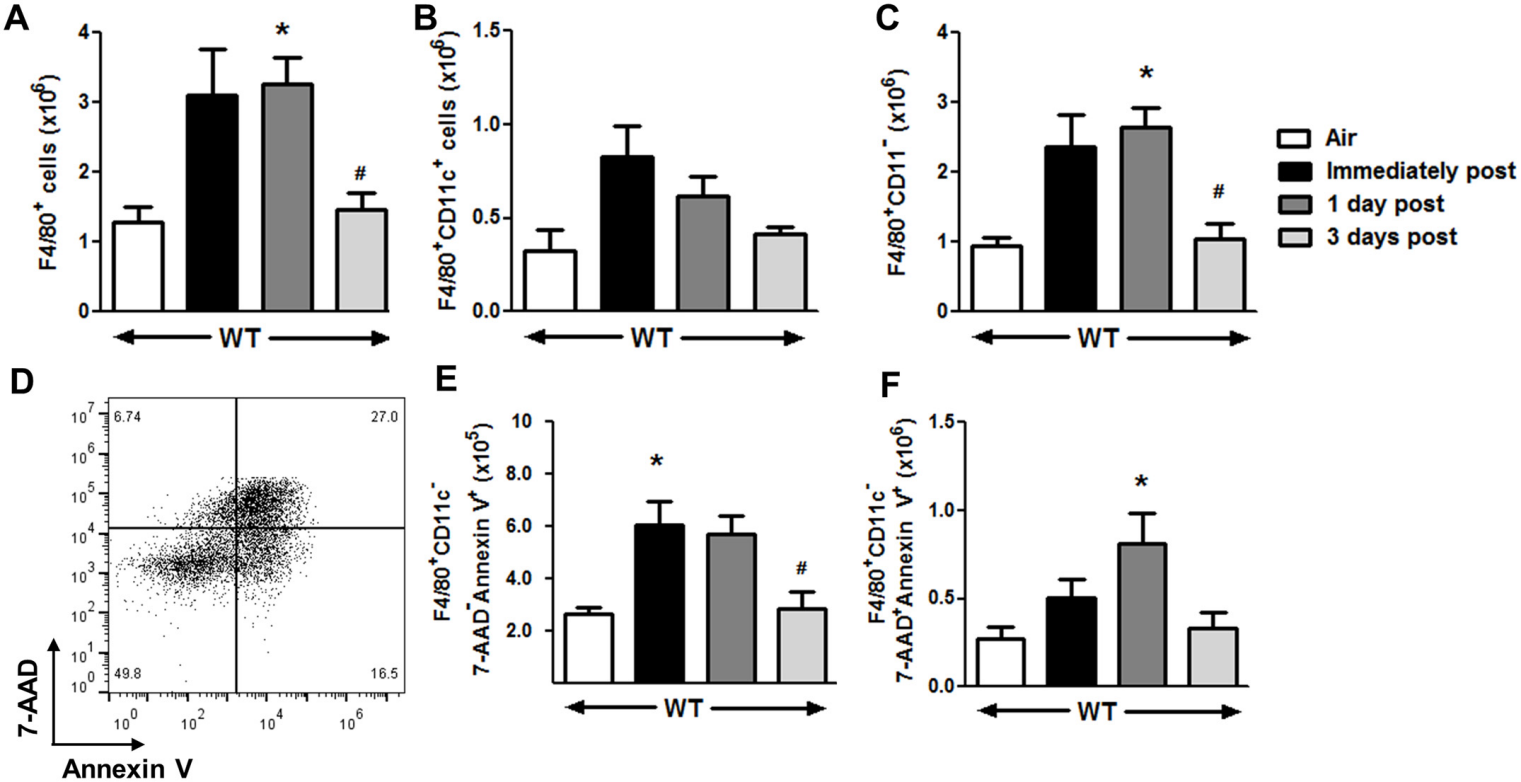
Lung apoptotic macrophages are elevated after O_3_ exposure. WT mice were exposed to either air or O_3_ (0.3 ppm for 72 h) and lungs were harvested either immediately or 1 or 3 days after cessation of O_3_ exposure. (A) Total macrophages, (B) Alveolar Macrophages, and (C) Interstitial macrophages assessed by flow cytometry. (D) Representative gating for apoptotic macrophages in a WT mouse studied 1 day after cessation of O_3_ exposure. (E) Early apoptotic interstitial macrophages and (F) late apoptotic interstitial macrophages in WT mice at various times after cessation of O_3_ exposure. Results are mean ± SEM for 4–6 mice per group. * p<0.05 versus air; # p<0.05 versus immediate post.

**Fig 6:**
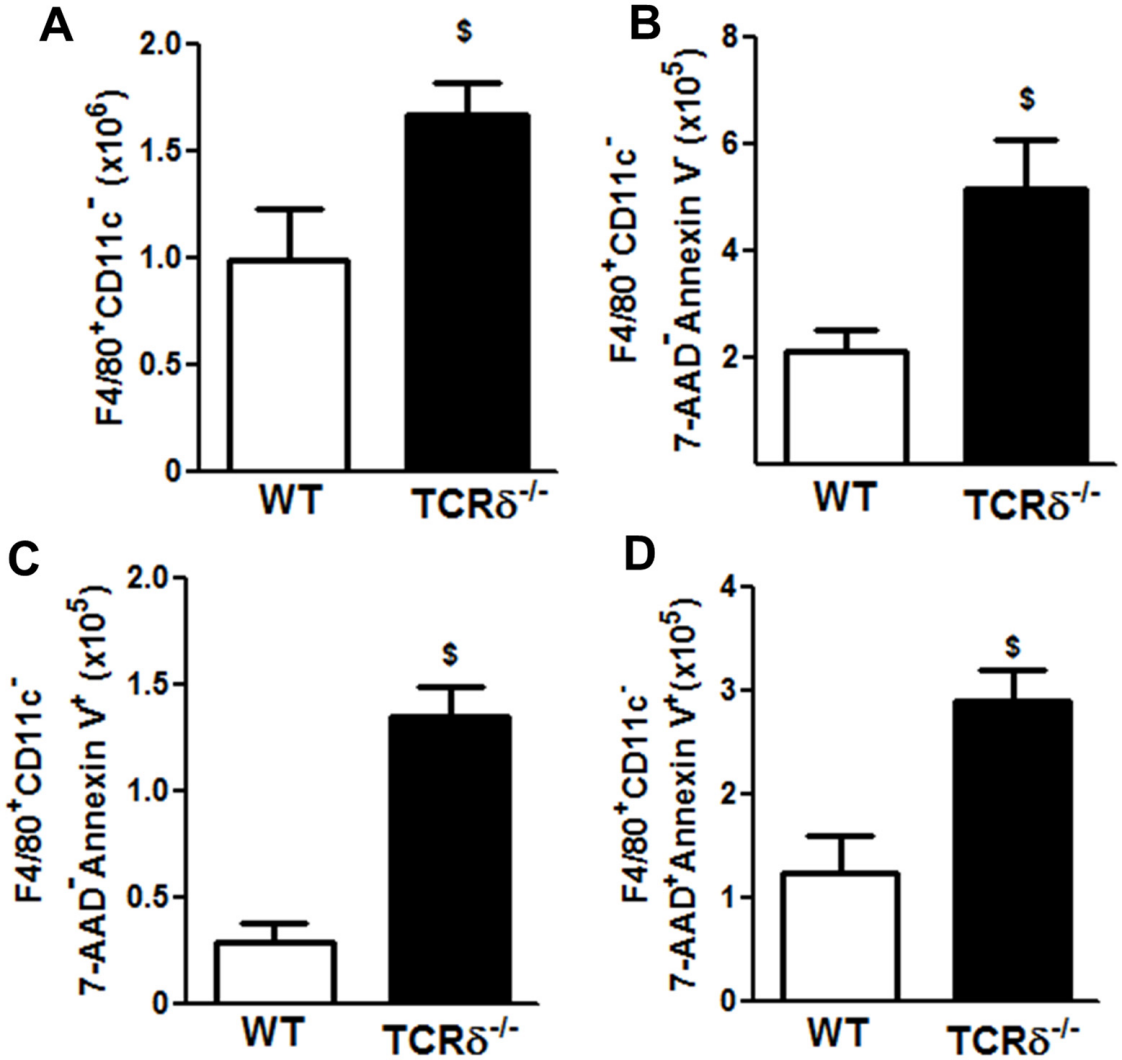
Macrophages accumulate in the lungs of TCRδ^-/-^ mice. (A) Total interstitial macrophages, (B) alive macrophages, (C) early apoptotic interstitial macrophages, and (D) late apoptotic interstitial macrophages in lungs of WT and TCRδ^-/-^ mice exposed to O_3_ for 72 h, and then transferred to room air and studied 3 days later. Results are mean ± SEM for 4–6 mice per group. $ p<0.05 versus WT mice.

We considered the possibility that reduced M2 polarization in TCRδ^-/-^ mice (Fig 1) would reduce clearance of apoptotic cells, including apoptotic macrophages, thus accounting for the sustained elevations of BAL macrophages after cessation of O_3_ exposure observed in TCRδ^-/-^ mice (Fig 4B). To address this possibility, we used flow cytometry to measure the number of apoptotic macrophages in the lung tissue of WT and TCRδ^-/-^ mice after O_3_ exposure. For these experiments, BAL was not performed. As described above, in WT mice, total lung macrophages (F4/80^+^ cells) were elevated in mice studied immediately after cessation of O_3_ exposure (Fig 1A). Increased total lung macrophages were sustained through 1 day after O_3_ exposure, and then declined at 3 days post O_3_ exposure (Fig 5A). This increase in lung macrophages was mostly due to an influx of F4/80^+^CD11c^-^ cells (interstitial macrophages [40]), which accounted for ~75% of the macrophages in the lung (compare Fig 5B and 5C). The number of early apoptotic (annexin V^+^/7-AAD^-^) CD11c^-^ macrophages (left upper quadrant in Fig 5D) peaked immediately post exposure and returned to air exposed levels within 3 days after O_3_ exposure (Fig 5E). Late apoptotic (annexin V^+^/7-AAD^+^) Cd11c^-^ macrophages (right upper quadrant in Fig 5D) peaked one day after O_3_ and returned to levels not different from air exposed mice within 3 days post O_3_ (Fig 5F). To determine if there were sustained elevations in apoptotic CD11c^-^ macrophages in TCRδ^-/-^ mice, we selected the 3 day post time point, as this was the time when apoptotic macrophages had returned to air exposed levels in WT mice. The number of interstitial macrophages (F4/80^+^CD11c^-^ cells) was significantly greater in TCRδ^-/-^ versus WT mice studied 3 days post O_3_ (Fig 6A). There were also greater numbers of non-apoptotic interstitial macrophages and of both early and late apoptotic in TCRδ^-/-^ versus WT mice (Fig 6B to 6D). We also found a trend towards an increase in alveolar macrophages (F4/80^+^CD11c^+^ cells) in TCRδ^-/-^ versus WT mice 3 days post O_3_ (data not shown) in TCRδ^-/-^ versus WT mice. In contrast the number of necrotic macrophages (upper left quadrant of Fig 5D) were similar between TCRδ^-/-^ and WT mice (data not shown).

### Role of γδ T cells in the resolution of O_3_-induced lung injury

**Fig 7:**
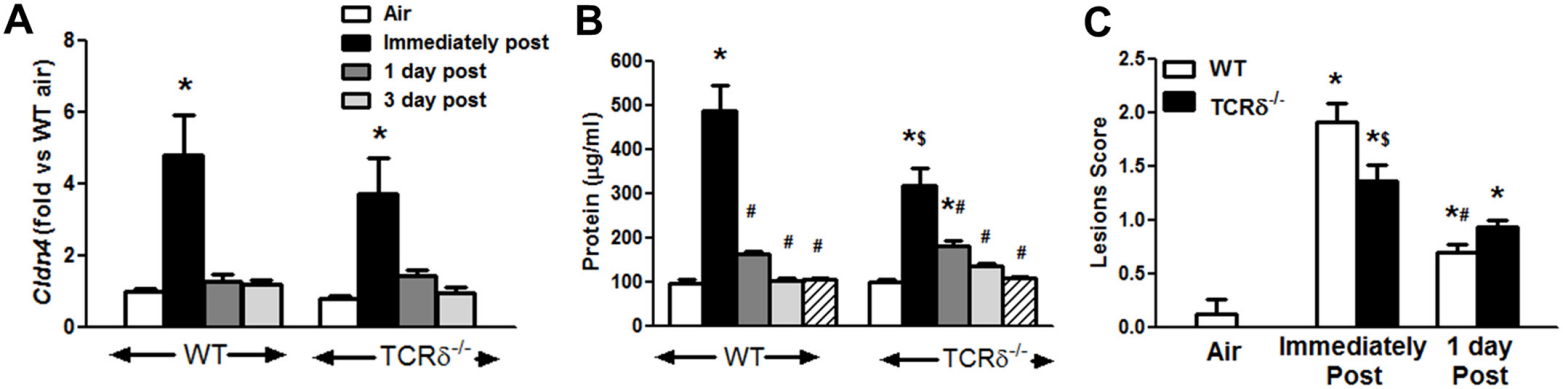
O_3_ induced injury. (A) pulmonary *Cldn4* mRNA abundance, (B) BAL protein, and (C) terminal bronchiolar lesions, scored as explained in the methods. Results are mean ± SE of 4–8 air exposed mice and 6–14 O_3_ exposed mice in each group. * p<0.05 versus air; # p<0.05 versus immediate post O_3_; $ p<0.05 versus WT mice.

Recovery from the effects of O_3_ requires repair of the damaged epithelium. In WT mice, BAL protein, an index of alveolar/capillary permeability reflecting damage to the lung epithelium [41], and *Cldn4*, a protein found in the tight junctions between pulmonary epithelial cells [42], were increased above air-exposed values immediately after cessation of O_3_ exposure, but not thereafter (Fig 7A and 7B), indicating very rapid resolution of changes in alveolar capillary permeability, likely reflecting restored formation of tight junctions. However, the lung architecture did not resolve as quickly. O_3_ causes terminal bronchiolar lesions [43,44], that reflect a combination of macrophage accumulation and epithelial hyperplasia. These lesions were scored from histological slides of lungs of WT and TCRδ^-/-^ mice (Fig 7C)[45], as described in methods. In WT mice, lesions were significantly greater in mice studied immediately after cessation of O_3_ exposure than in air exposed mice, but within 1 day of cessation of exposure, the lesion score declined significantly (Fig 7C). O_3_ also increased lesions in TCRδ^-/-^ mice though the score immediately post exposure was lower than in WT mice (Fig 7C). However, in contrast to WT mice, there was no reduction in the lesion score 1 day after compared to immediately after cessation of O_3_ exposure (Fig 7C).

## Discussion

We have previously reported that γδ T cells contribute to the pulmonary recruitment of neutrophils and macrophages that occurs after subacute O_3_ exposure in mice [28]. We now report that γδ T cells are also required for the induction of M2 macrophages after subacute O_3_ (Fig 1), likely as a result of the ability of γδ T cells to produce IL-17A (Fig 3). Consequently, after cessation of O_3_ exposure, clearance of apoptotic cells and resolution of pulmonary inflammation are delayed in TCRδ^-/-^ mice that lack γδ T cells (Figs 4 and 6) after O_3._

Our data indicated that in WT mice, M2 macrophages were induced by subacute O_3_ exposure, with levels peaking between 48 and 72 hours of exposure (Fig 1B). M2 gene expression also increased, peaking at 48 hours of exposure (Fig 1C to 1E). The slight difference in time course between M2 macrophages assessed by flow cytometry and M2 gene expression may result from reductions in M2 gene expression that occur after macrophages polarize to M2b and M2c [46,47]. M2 macrophages are also induced in the lungs by acute O_3_ exposure (2 ppm for 3 h) [15] and by other insults that induce oxidative stress in the lungs [48]. In contrast, compared to WT mice, in TCRδ^-/-^ mice we observed decreased numbers of M2 macrophages (Fig 1H) and decreased pulmonary mRNA abundance of M2 genes after subacute O_3_ (Fig 1C to 1E and Fig 2A to 2C), indicating that γδ T cells are required for induction of the M2 phenotype. M1 macrophages also increased after O_3_ exposure, but were not affected by TCRδ deficiency (Fig 1G). However, BAL TNFα, was higher in TCRδ^-/-^ versus WT mice, at least during the first 48 hours of exposure (Fig 1I). M1 macrophages are a likely source of this TNFα [36] and the increase in BAL TNFα could thus reflect an increase in M1 activity in the TCRδ^-/-^ mice versus WT mice.

Given the close apposition of γδ T cells and macrophages within the lungs and airways [17], it is certainly possible that factors released from γδ T cells after O_3_ might have the capacity to polarize macrophages. For example, type 2 cytokines, including IL-13, promote M2 skewing in macrophages [49], and γδ T cells have the capacity to produce IL-13 [50]. However, RT-qPCR indicated that O_3_-induced changes in pulmonary *Il13* mRNA abundance were essentially similar in WT and TCRδ^-/-^ mice (Fig 2D). In addition to IL-13, IL-17A can interact with IL-10 to induce macrophage polarization towards an efferocytic M2c phenotype that promotes inflammatory cell clearance [13]. We have previously reported that γδ T cells in the lungs of O_3_-exposed mice produce IL-17A [27,28]. and that increases in pulmonary *Il17a* mRNA abundance induced by subacute O_3_ exposure are absent in TCRδ^-/-^ mice [28], suggesting that the role of γδ T cells in the M2 polarization observed after subacute O_3_ may be related to the ability of γδ T cells to release IL-17A. Indeed, our data indicated no evidence of *Il17a* mRNA expression in TCRδ^-/-^ mice either immediately after cessation of O_3_ exposure or at any time over the next 3 days (Fig 2E and 2F). Furthermore, when we blocked IL-17A with anti-IL-17A in WT mice, we found that the induction of M2 macrophages was attenuated (Fig 3A and 3B) and the activity of the M1 macrophages was increased (Fig 3C). IL-17A^+^ γδ T cells are also required for the resolution of eosinophilic inflammation after allergen challenge in mice [25] and IL-17A is also protective in several mouse models of colitis [51,52]. The mechanistic basis for these protective effects of IL-17A has not been established, but our data suggest that they may be the result of the ability of IL-17A to promote polarization of macrophages to an M2c phenotype, thereby permitting clearance of dead and dying inflammatory cells.

In WT mice, significant increases in BAL neutrophils and macrophages were observed immediately after cessation of O_3_ exposure, but both cell types declined significantly within 1 day of the termination of exposure and returned to levels not different from air exposed controls within 3 days (Fig 4A and 4B). These data are consistent with the results of Kleeberger et al [8], who used the same O_3_ exposure regimen and also reported resolution of inflammation within 3 days of the termination of exposure in WT mice. Although the initial increases in BAL neutrophils and macrophages induced by O_3_ were significantly lower in TCRδ^-/-^ than WT mice, as described previously [28], the return of these cells towards normal air-exposed values after cessation of O_3_ was slower in TCRδ^-/-^ versus WT mice (Fig 4). In TCRδ^-/-^ mice, BAL neutrophils actually *increased* transiently after cessation of O_3_ (Fig 4A), and even 3 days after cessation of exposure BAL macrophages had not declined from values reached immediately after exposure (Fig 4B). Similarly, the number of lung macrophages (F4/80^+^) (the majority of which are interstitial macrophage (data not shown)) were similar between the WT and TCRδ^-/-^ mice immediately after exposure (Fig 1F), but by 3 days after cessation of exposure there were more pulmonary interstitial macrophages in the TCRδ^-/-^ vs the WT mice (Fig 6A). While it is conceivable that the observed genotype-related differences in the time course of changes in inflammatory cells after cessation of O_3_ exposure (Fig 4A and 4B) represent delayed induction of inflammation rather than reduced resolution of inflammation in the TCRδ^-/-^ versus WT mice, however our data provide little support for such a hypothesis, since other non-cellular inflammatory parameters decreased rapidly once the O_3_ exposure was terminated, even in TCRδ^-/-^ mice (Fig 4C and 4D). To separate effects of γδ T cells on the *resolution* of inflammation from their effects on the *induction* of inflammation, the ideal design would have been to ablate γδ T cells immediately after exposure to O_3_, for example with anti-TCRδ antibodies [25], so that the induction of inflammation was not impacted. Unfortunately, the time course of resolution of inflammation after O_3_ was sufficiently quick that it did not permit such a design: most inflammatory parameters had returned to air-exposed levels within 1 to 3 days after O_3_ cessation, and eliminating γδ T cells with antibodies could not be achieved in this time frame. Instead, we used TCRδ^-/-^ mice, in which both the induction and resolution of inflammation were impacted. The use of TCRδ^-/-^ mice to study the initiation and resolution of inflammation has also been employed in other disease models with similar results [19,26,28,53]. In addition, the observation that in the lungs of TCRδ^-/-^ mice studied 3 days after cessation of exposure, most of the macrophages were in an apoptotic state (Fig 6B to 6D), suggests that lack of clearance rather than continued recruitment accounts for greater numbers of macrophages in the lungs of TCRδ^-/-^ versus WT mice at this time (Fig 6A). These findings are similar to the results of Ponomarev et al [53] who reported reduced numbers of macrophages in the central nervous system of TCRδ^-/-^ versus WT mice during *induction* but greater numbers of macrophages during *resolution* of inflammation in a model of experimental autoimmune encephalomyelitis (EAE). Similarly, Kirby et al [26] reported greater numbers of lung macrophages in TCRδ^-/-^ versus WT mice during the resolution phase of *S*. *pneumoniae*-induced pulmonary inflammation.

In addition to an accumulation of apoptotic macrophages, the number of non-apoptotic (alive) macrophages were also increased in the TCRδ^-/-^ versus WT mice 3 days after cessation of O_3_ (Fig 6B). γδ T cells can promote death of activated macrophages via their ability to recognize heat shock proteins expressed by these activated cells [54]. In addition, γδ T cells express FASL and can induce the apoptosis of macrophages [55] after bacterial infections. Our data suggests that after O_3_ γδ T cells have a dual role in the clearance of macrophages, namely to induce their apoptosis and to induce the polarization of macrophages to M2 phenotype which then clear the apoptotic cells.

As discussed above, our data suggest that the delayed clearance of inflammatory cells observed in TCRδ^-/-^ mice after cessation of O_3_ exposure (Fig 4A and 4B; Fig 6A) is at least in part the result of the reduced M2 macrophage polarization observed in the TCRδ^-/-^ mice (Figs 1 and 2). Efferocytic M2c macrophages are required for phagocytosis of apoptotic cells, including neutrophils and macrophages [56] and our data indicate that many of the macrophages that remained in the lungs after cessation of O_3_ exposure were indeed apoptotic (Fig 6C and 6D). The observations that both early and late apoptotic interstitial macrophages (Fig 6C and 6D) were greater in TCRδ^-/-^ than WT mice 3 days post of cessation of O_3_ suggests that apoptotic macrophages are cleared less effectively in TCRδ^-/-^ mice. Indeed most of the increased total interstitial macrophages in TCRδ^-/-^ mice observed 3 days after cessation of O_3_ exposure consisted of apoptotic cells (compare Fig 6A to Fig 6C and 6D). Such results are consistent with the lack of M2 macrophages observed in O_3_-exposed TCRδ^-/-^ mice (Figs 1 and 2).

In addition to clearing apoptotic macrophages, M2 macrophages are important in the repair of the damaged tissue [37]. In this respect, reduced induction of M2 macrophages in TCRδ^-/-^ mice (Figs 1 and 2) is consistent with delayed restoration of the normal architecture of the lung in TCRδ^-/-^ mice (Fig 7C). Terminal bronchiolar lesions, which in part reflect injury-induced changes to epithelial cells, resolved rapid in WT mice: lesions were reduced to only a third of their peak value within 1 day of cessation of exposure. In contrast, in TCRδ^-/-^ mice, lesions were still unchanged from peak values 1 day after cessation of exposure.

While our data strongly suggest that lack of M2 macrophages capable of phagocytosing apoptotic inflammatory cells accounted for the delayed clearance of inflammatory cells observed in TCRδ^-/-^ versus WT mice, we cannot rule out the possibility that other factors also contributed to the role of γδ T cells in these events. For example, as discussed above γδ T cells can induce macrophage apoptosis and γδ T cells are found in close proximity to the pulmonary epithelium [17] and can secrete epithelial growth factors [57]. Hence, it is also possible that loss of such effects in TCRδ^-/-^ mice might translate into altered secretion of pro-resolving molecules that contribute to the resolution of inflammation, many of which are derive from the epithelium [58].

In summary, our data indicate that γδ T cells are required for induction of M2 macrophages and consequent inflammatory cell clearance and repair of the epithelial layer in mice after subacute O_3_ exposure. These data have potentially important implications for public health, especially for pollutant-exposed immune-compromised individuals who have dysfunctional T cells.
